# Generation of mutant pigs by lipofection-mediated genome editing in embryos

**DOI:** 10.1038/s41598-021-03325-5

**Published:** 2021-12-13

**Authors:** Maki Hirata, Manita Wittayarat, Zhao Namula, Quynh Anh Le, Qingyi Lin, Koki Takebayashi, Chommanart Thongkittidilok, Taro Mito, Sayuri Tomonari, Fuminori Tanihara, Takeshige Otoi

**Affiliations:** 1 Faculty of Bioscience and Bioindustry, Tokushima University, Tokushima, Japan; 2 Bio-Innovation Research Center, Tokushima University, Tokushima, Japan; 3 Faculty of Veterinary Science, Prince of Songkla University, Songkhla, Thailand; 4 College of Coastal Agricultural Sciences, Guangdong Ocean University, Guangdong, China; 5 Present Address: Center for Development of Advanced Medical Technology, Jichi Medical University, Tochigi, Japan

**Keywords:** Genetic engineering, Biological techniques, Biotechnology, Embryogenesis

## Abstract

The specificity and efficiency of CRISPR/Cas9 gene-editing systems are determined by several factors, including the mode of delivery, when applied to mammalian embryos. Given the limited time window for delivery, faster and more reliable methods to introduce Cas9-gRNA ribonucleoprotein complexes (RNPs) into target embryos are needed. In pigs, somatic cell nuclear transfer using gene-modified somatic cells and the direct introduction of gene editors into the cytoplasm of zygotes/embryos by microinjection or electroporation have been used to generate gene-edited embryos; however, these strategies require expensive equipment and sophisticated techniques. In this study, we developed a novel lipofection-mediated RNP transfection technique that does not require specialized equipment for the generation of gene-edited pigs and produced no detectable off-target events. In particular, we determined the concentration of lipofection reagent for efficient RNP delivery into embryos and successfully generated *MSTN* gene-edited pigs (with mutations in 7 of 9 piglets) after blastocyst transfer to a recipient gilt. This newly established lipofection-based technique is still in its early stages and requires improvements, particularly in terms of editing efficiency. Nonetheless, this practical method for rapid and large-scale lipofection-mediated gene editing in pigs has important agricultural and biomedical applications.

## Introduction

Genetically engineered pigs are important animal models for biomedical research owing their anatomical and physiological similarities to humans^1,2^. The first genetic modification of pigs was achieved over 35 years ago by the pronuclear microinjection of exogenous DNA into single-cell embryos^3^. Since then, several strategies have been developed to generate a reliable and efficient method for the introduction of genetic alterations into porcine embryos; these include sperm-mediated transfection^4^, somatic cell nuclear transfer^5^ and oocyte transduction via viral vectors^6^.

Targeted nucleases are powerful tools for gene modification with high precision in pigs^7^. Clustered regularly interspaced short palindromic repeats (CRISPR) and CRISPR-associated protein 9 (Cas9), which is composed of a guide RNA (gRNA) and a Cas9 nuclease, are widely used for efficient and versatile gene editing in various organisms by simply specifying a 20-nucleotide targeting sequence within a gRNA^8–10^. At present, genetically modified pigs are typically established by somatic cell nuclear transfer using gene-modified somatic cells and the direct introduction of gene editors into the cytoplasm of zygotes and embryos via microinjection or electroporation^7,11^. However, these conventional methods require expensive equipment and sophisticated techniques. Simple, rapid, and repeatable methods for highly efficient gene modification are needed.

Lipofection, defined as lipid-mediated gene transfer, involves the introduction of foreign genes into mammalian cells using lipophilic reagents that increase the cellular uptake of polynucleotides^12,13^. Without the use of specialized equipment, Cas9 protein and gRNA can be co-delivered into various mammalian cells using the lipofection mechanism^14–16^. Recently, we successfully demonstrated lipofection-mediated gene editing in in vitro fertilized porcine zygotes and embryos without zona pellucida (ZP)^17^. Although the efficiency was insufficient, lipofection-mediated gene editing during embryogenesis can substantially improve the value of pig resources as experimental animals, particularly in unequipped laboratories. However, lipofectamine, a common reagent used to introduce DNA into cells, can be toxic and induce cell death at rates of approximately 35–65% under certain conditions^18^. Therefore, careful investigations are needed to achieve both high viability and high gene-editing efficiency in lipofection-treated embryos and to demonstrate developmental competence until the fetal stage or delivery. Here, for the first time, we report the generation of genetically modified pigs by the CRISPR/Cas9 system via the lipofection-mediated introduction of Cas9-gRNA ribonucleoprotein complexes (RNPs) into porcine embryos.

## Results

We have previously optimized the timing of lipofection treatment for the introduction of the CRISPR/Cas9 system into ZP-free embryos, demonstrating that the treatment of 1- to 8-cell stage embryos at 29 h from the start of in vitro fertilization (IVF) for 5 h yielded a high gene editing efficiency^17^. In this study, jetCRISPR (Polyplus-transfection, Illkirch-Graffenstaden, France) was used as RNP transfection reagent for CRISPR/Cas9-mediated gene editing in mammalian cells^19,20^. We targeted the myostatin (*MSTN*) gene, which encodes a negative regulator of muscle growth and whose disruption typically results in increased skeletal muscle mass^21,22^. Although knockout of *MSTN* gene leads to some defects, such as lameness and hindlimb weakness depending on targeting exons and pig breed^23,24^, the double muscle phenotype and lack of lethal effects on offspring are valuable for evaluating successful gene modification.

### Determination of the jetCRISPR concentration for gene editing of MSTN

**Figure 1:**
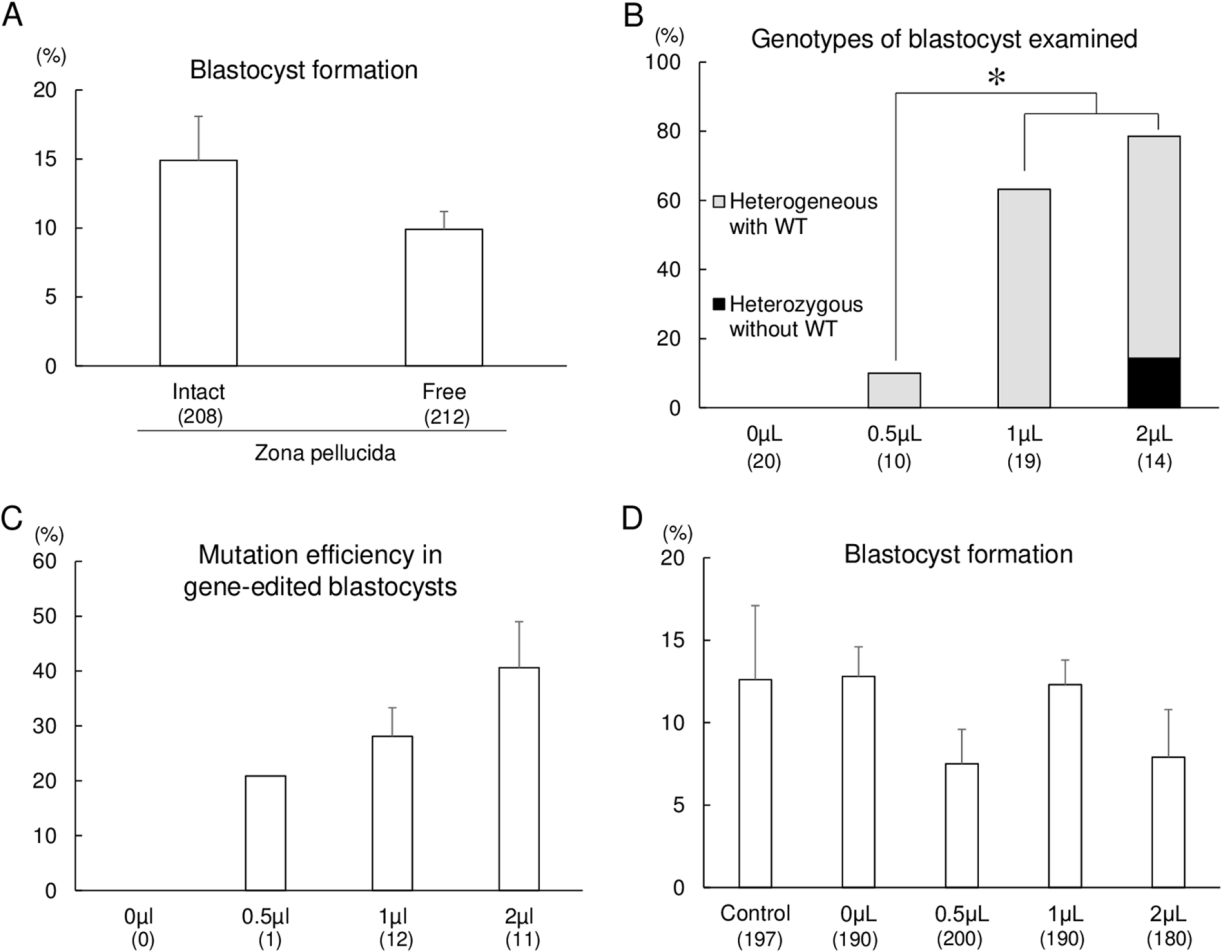
Optimization of the jetCRISPR concentration for gene editing of *MSTN*. (**A**) Blastocyst formation rates of ZP-intact and ZP-free embryos without RNP transfection. (**B**) Frequency of gene editing in the target regions of blastocysts derived from the embryos treated with jetCRISPR, Cas9 protein, and gRNA. Gene editing of blastocysts was determined by Sanger sequencing and TIDE. The percentage of blastocysts with gene editing was defined as the ratio of the number of gene edited blastocysts to the total number of blastocysts examined. (**C**) Mutation efficiency in gene-edited blastocysts. Editing efficiency was defined as the proportion of indel mutation events in blastocysts carrying mutations. Heterozygous without WT: blastocysts carrying multiple types of editing but no WT sequences, Heterogeneous with WT: blastocysts carrying mosaic mutation or heterozygous mutation carrying more than one type of mutation and the WT sequence, and monoallelic mutation. (**D**) Blastocyst formation rates of embryos treated with various concentrations of jetCRISPR. Each bar represents the mean ± SEM. Four replicate trials were carried out and the numbers in parentheses indicate the total number of oocytes (**A**,**D**) and examined blastocysts (**B**,**C**). Percentages of blastocysts carrying mutations in target genes were analyzed using chi-squared tests (**B**). *p < 0.05.

First, we determined the concentration of the RNP transfection reagent with respect to the efficiency of gene editing. 1- to 8-cell stage embryos collected at 29 h from the start of IVF were freed from the ZP using actinase-E (Kaken-Seiyaku Corp., Tokyo, Japan) and incubated for 5 h in 500 µL of culture medium (PZM-5; Research Institute for the Functional Peptides Co., Yamagata, Japan) containing 10 ng/μL gRNA (Alt-R CRISPR crRNAs and tracrRNA from IDT; Integrated DNA Technologies, Coralville, IA, USA), 30 ng/μL Cas9 protein (Guide-it Recombinant Cas9; Takara Bio, Shiga, Japan), and 0.5, 1, or 2 µL of jetCRISPR. After in vitro culture for an additional 6 days, the blastocyst formation rate and gene-editing efficiency in the resulting blastocysts were evaluated. As a control for the analysis of embryonic development, some ZP-free embryos without RNP transfection were cultured. The blastocyst formation rate of ZP-intact and ZP-free embryos are statistically same, indicating that the removal of ZP after IVF had no harmful effect on embryonic development (Fig. 1A). Blastocyst genotypes were classified as homozygous editing (including only a single type of editing), heterozygous editing without WT (including multiple types of editing but carrying no WT sequences), heterogeneous editing with WT (including mosaic or heterozygous mutation carrying more than one type of mutation and the WT sequence, and monoallelic mutation), or WT (carrying only the WT sequence). As it is difficult to distinguish between heterozygous and mosaic embryos based on the results of Sanger sequencing, we classified blastocysts that have both mutant and WT sequences as heterozygous in this study.

Indel mutations in the target region of the *MSTN* gene were detected in the group treated with 0.5–2 µL of jetCRISPR (Fig. 1B). However, homozygous mutant embryos that have only one type of edited sequence were not detected, and heterozygous editing without WT was only detected in blastocysts derived from embryos treated with 2 µL of jetCRISPR. The total gene editing rates, including rates of heterogeneous with WT and heterozygous editing without WT, were significantly higher (*p* < 0.05) in blastocysts from embryos treated with 1 and 2 µL than with 0.5 µL of jetCRISPR. The frequency of indel mutation events in the gene-edited blastocysts was quantified using tracking of indels by decomposition (TIDE)^25^ and is shown in Fig. 1C. Frequency of indel mutation events in blastocysts from embryos treated with 1 and 2 µL of jetCRISPR was statistically same, while indel mutation events from embryos treated with 0.5 µL jetCRISPR could not be compared with other groups due to low sample number. No significant differences in the blastocyst formation rate were observed among groups treated with different volumes of jetCRISPR (Fig. 1D).

### Confirmation of gene editing with RNP transfection targeting five different genes

**Figure 2:**
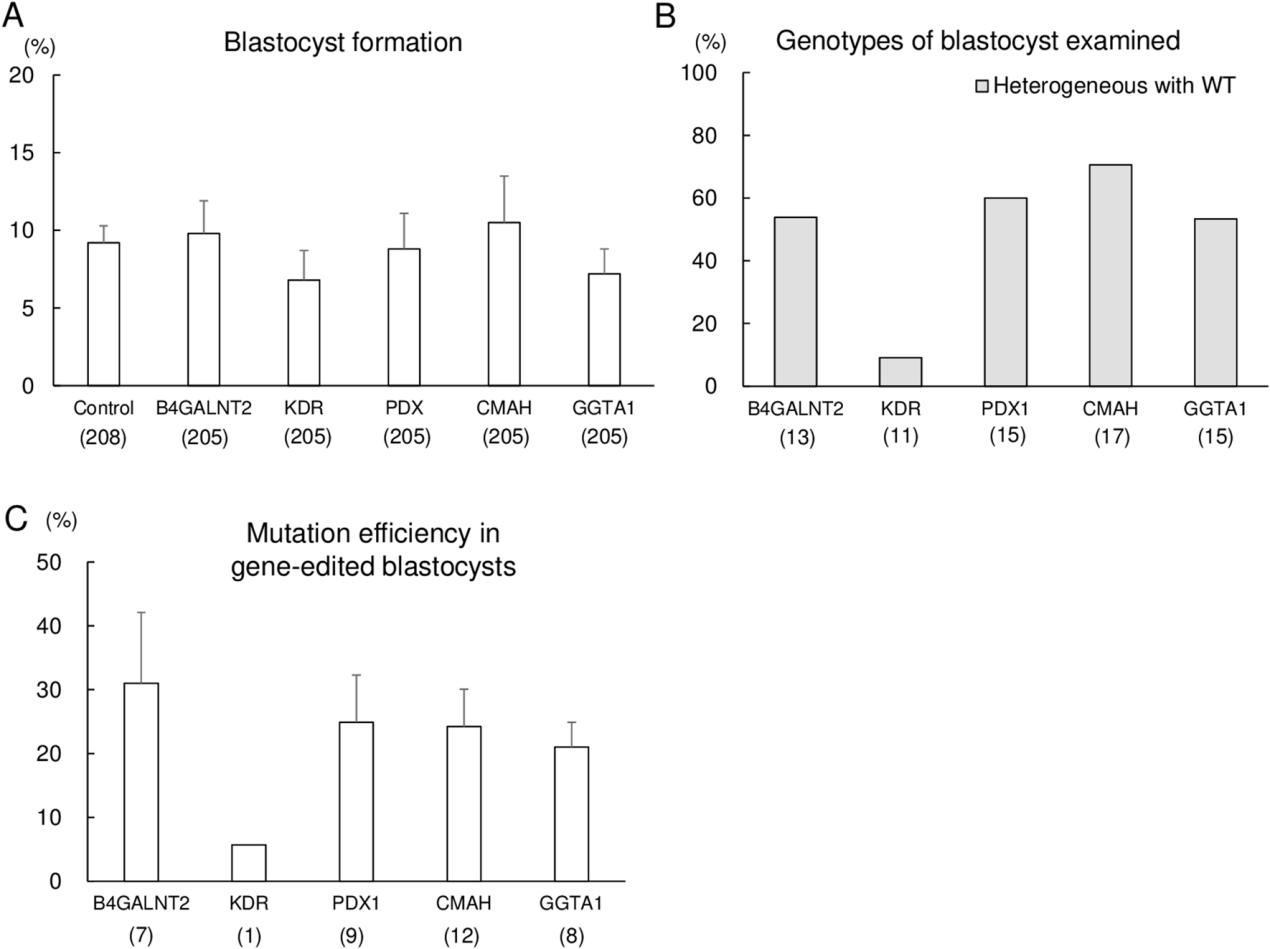
Confirmation of gene editing with jetCRISPR targeting five different genes. (**A**) The blastocyst formation rate of jetCRISPR treated embryos. Each bar represents mean ± SEM. (**B**) Frequency of gene editing in the target regions of blastocysts derived from the embryos treated with jetCRISPR, Cas9 protein, and gRNAs. Gene editing of the blastocysts was determined by Sanger sequencing and a TIDE analysis. The percentage of blastocysts with gene editing was defined as the ratio of the number of gene edited blastocysts to the total number of blastocysts examined. (**C**) Mutation efficiency in gene-edited blastocysts. Editing efficiency was defined as the proportion of indel mutation events in blastocysts carrying mutations. Heterogeneous with WT: blastocysts carrying mosaic mutation or heterozygous mutation carrying more than one type of mutation and the WT sequence, and monoallelic mutation. Four to five replicate trials were carried out and the numbers in parentheses indicate the total number of oocytes (**A**) and examined blastocysts (**B**,**C**).

To evaluate the versatility of jetCRISPR-mediated transfection in porcine embryos, we evaluated the editing efficiency of the CRISPR/Cas9 system targeting various genes (*B4GALNT2*, *KDR*, *PDX1*, *CMAH*, and *GGTA1*) related to xenoantigen biosynthesis and organ development, a key process for transplantation of organs regenerated using xenogeneic stem cells in pigs. We confirmed the gene editing efficiency of gRNA targeting *KDR* by electroporation, and selected KDR#1 for use in the jetCRISPR-mediated transfection (Supplementary Fig. S1). Efficiency of gRNAs targeting *B4GALNT2*, *PDX1*, *CMAH*, and *GGTA1*was evaluated by electroporation-mediated gene editing in our previous study^26–29^. Briefly, 500 µL of PZM-5 containing 10 ng/μL gRNA, 30 ng/μL Cas9 protein, and 2 µL of jetCRISPR reagent was used for lipofection-mediated gene editing. As a control for the analysis of embryonic development, some ZP-free embryos without lipofection-mediated gene editing were cultured. The rates of blastocyst development were not affected by lipofection treatment targeting different sites (Fig. 2A). Homozygous and heterozygous mutation without WT were not detected in the resulting blastocysts, and only blastocysts carrying heterogeneous mutation with WT were detected for each gene (Fig. 2B). Frequency of indel mutation events in the gene-edited blastocysts was consistent with the result of gene editing rates shown in Fig. 2B (Fig. 2C). There were no significant differences in frequency of indel mutation events among the targeted genes. As 2 µL of jetCRISPR was effective for the introduction of the CRISPR/Cas9 system, this volume was used to produce *MSTN*-edited pigs via RNP transfection into porcine embryos.

### Generation of MSTN-edited pigs derived from lipofection-treated ZP-free embryos

**Figure 3:**
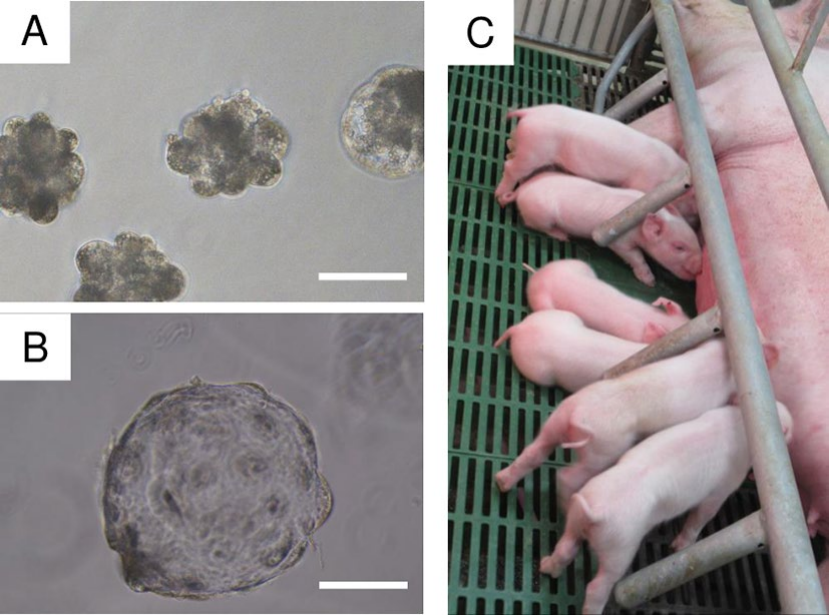
Photographs of lipofection-treated ZP-free embryos (day 4) (**A**), ZP-free blastocyst (Day 7) (**B**), and delivered piglets (**C**). The scale bar in each panel represents 100 μm.

Figure 3A,B show the lipofection-treated ZP-free embryos. Blastocysts treated with jetCRISPR with RNP targeting *MSTN* were transferred into the uterus of a recipient gilt. Thirty early blastocysts were transferred to a single recipient gilt resulting in pregnancy and the birth of 9 piglets (Fig. 3C). Two piglets (#1 and #9) were stillborn, and one piglet (#2) was crushed by the sow and died soon after the accident.

**Figure 4:**
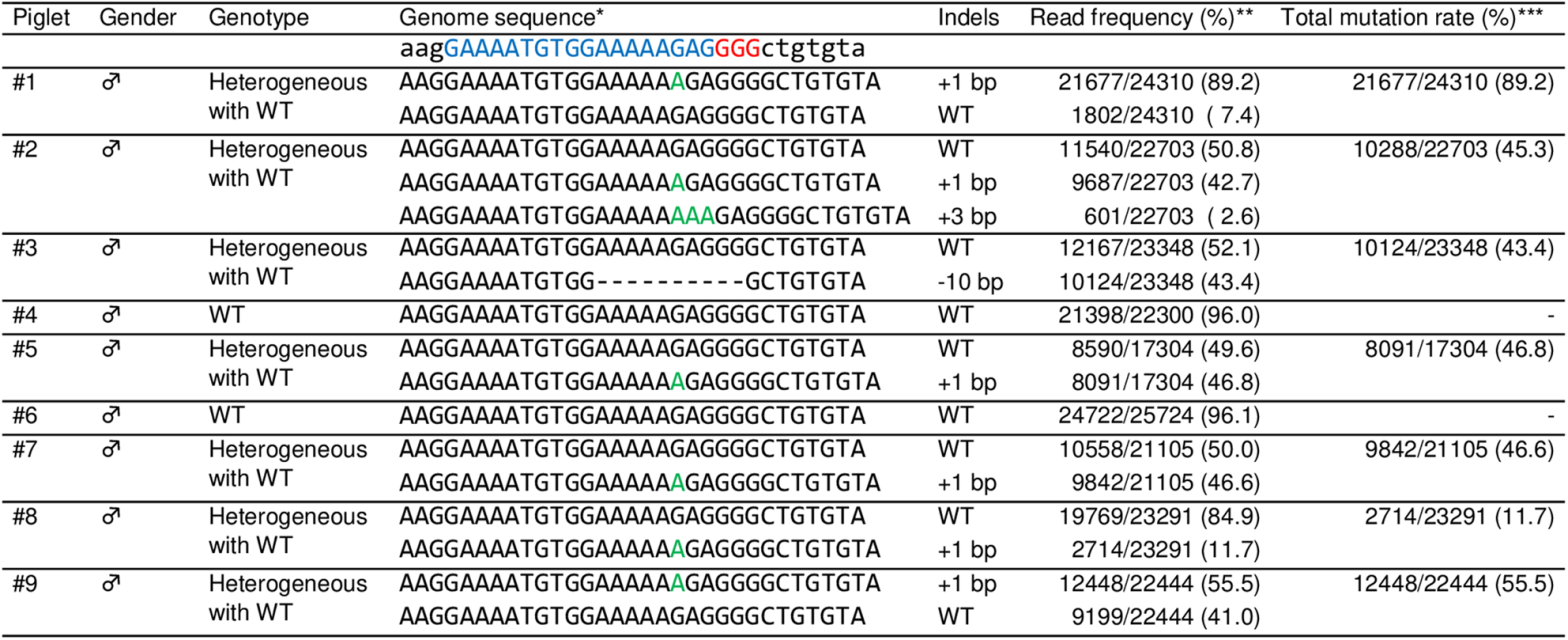
Deep sequencing analysis of the *MSTN* target region in delivered piglets. *Nucleotides in blue and red represent the target sequences and PAM sequences of gRNA, respectively. Nucleotides in green represent inserted nucleotids. **The read frequency was defined as the ratio of the number of reads to the total number of aligned read. ***The total mutation rate was defined as the ratio of the total number of modified reads to the total number of aligned reads. *WT* wild-type; ♂, male.

**Figure 5:**
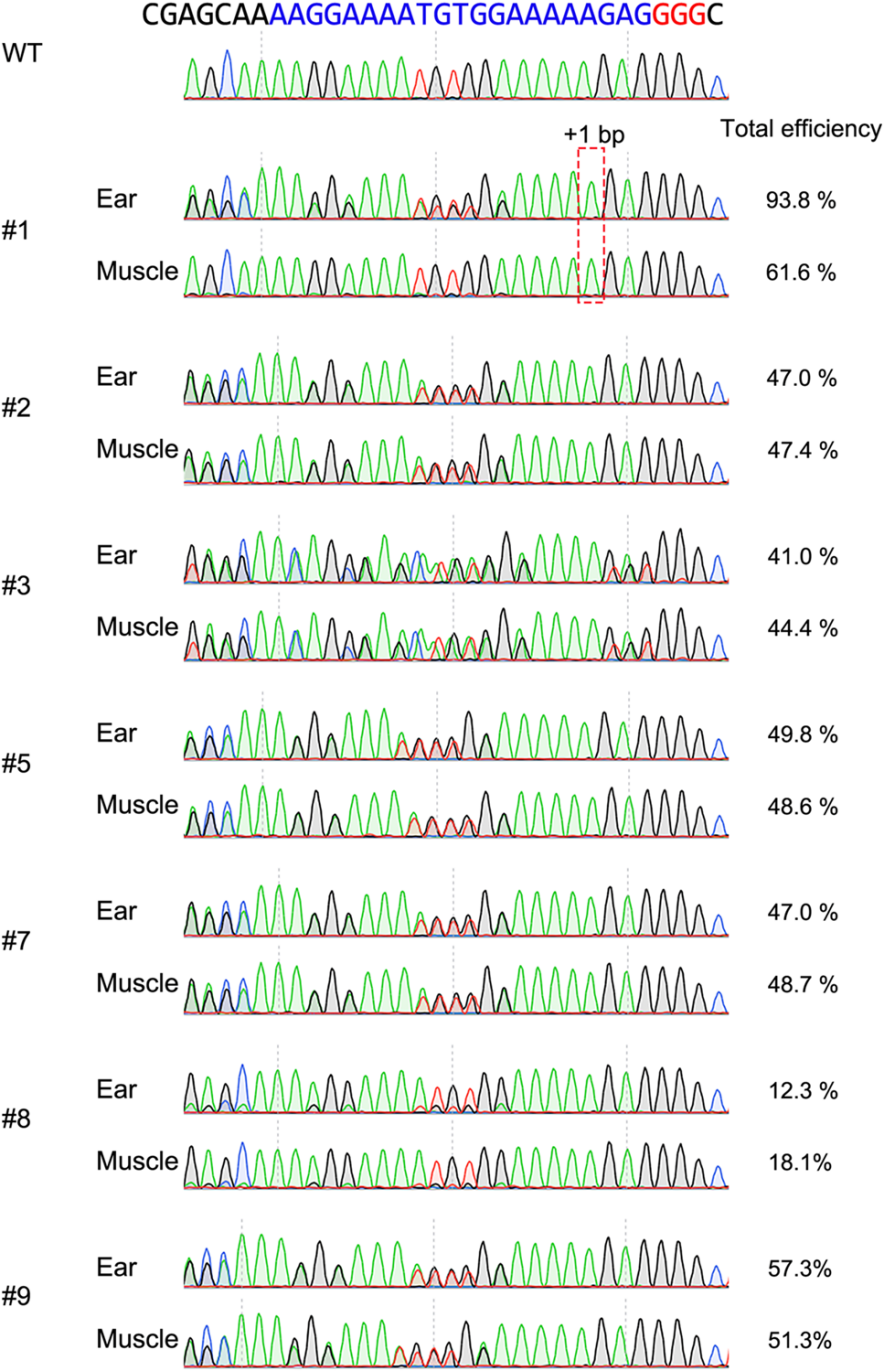
Sanger sequencing analysis of muscle and ear tissues derived from *MSTN*-mutant and wild-type pigs, and their total mutation frequency of indel mutations. Total efficiency was defined as the frequency of indel mutations decomposed from Sanger sequence data by TIDE analysis.

**Figure 6:**
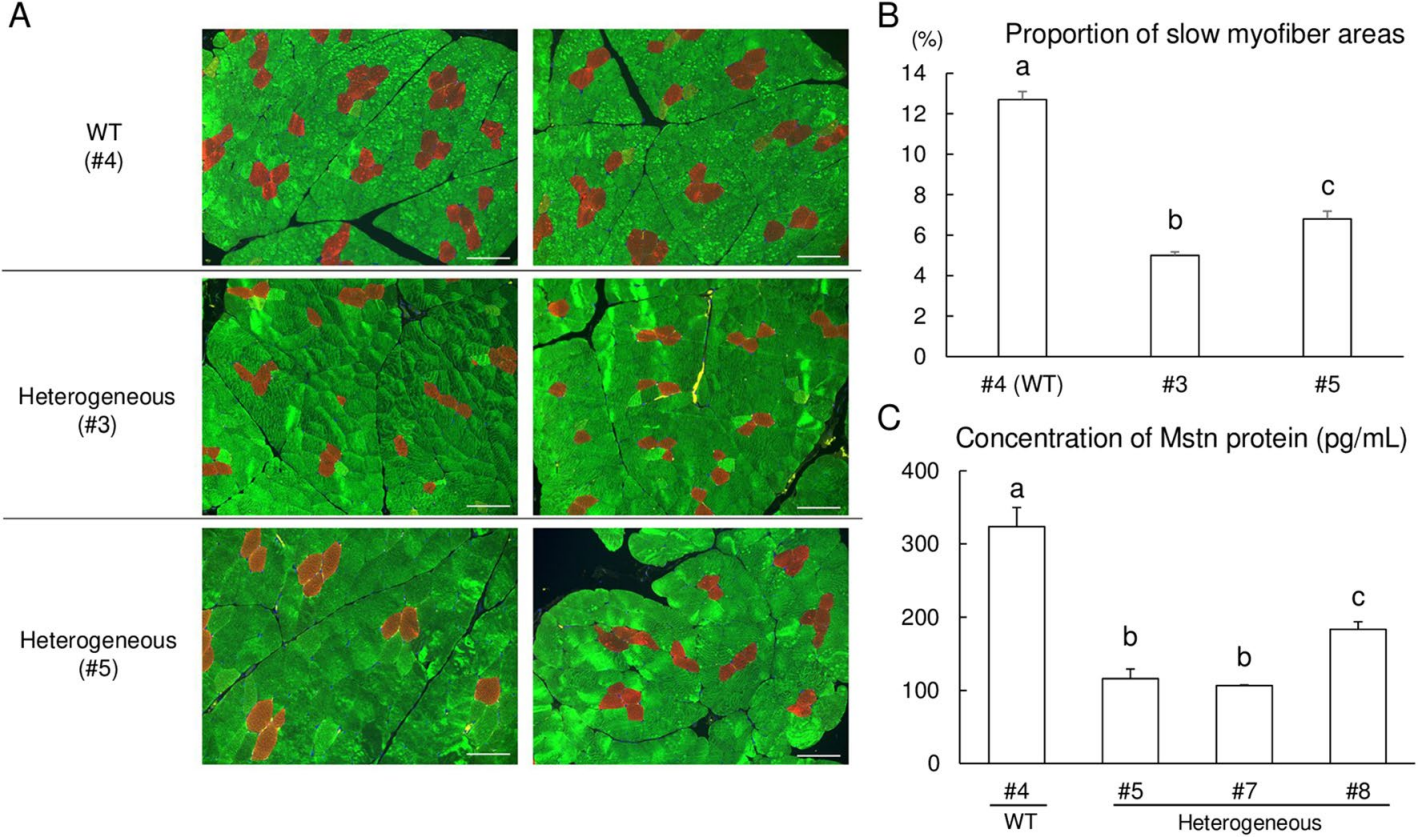
Immunohistochemical assessment and quantification of MSTN protein concentration of wild-type (WT) and *MSTN* heterogeneous mutant piglets. (**A**) The longissimus dorsi muscles biopsies derived from WT (#4) and mutant piglets (#3 and #5) were immunohistochemically stained for slow (red) and fast (green) skeletal muscle myosin. The scale bar in each panel represents 100 μm. (**B**) Proportion of slow myofibers in longissimus dorsi muscle tissues. The slow myofiber areas were calculated as percentages from seven images after immunofluorescence staining for slow and fast type muscle fiber markers in longissimus dorsi muscle tissues obtained from 40-day-old piglets. WT, wild-type. Each bar represents a mean ± SEM. ^a–c^*p* < 0.05. (**C**) Comparison of MSTN protein concentrations. Equal concentrations (1.0 mg mL^−1^) of total protein extracts obtained from the longissimus dorsi muscle of the wild type (WT; #4) and *MSTN*-mutant pigs (#5, #7 and #8) were used for ELISA. Each sample was assessed in quadruplet (n = 4), and the data are expressed as the mean ± SEM. ^a–c^*p* < 0.05.

Genomic sequences at the target region in *MSTN* of all delivered piglets were analyzed by deep sequencing (Fig. 4). Genomic DNA was extracted from ear biopsy samples, and the target regions were amplified and indexed. Pooled amplicons were sequenced using an Illumina Miseq platform, and the sequencing results were analyzed using CRISPResso2. No reads with indel mutations or base substitutions at the gRNA targeted region were detected in two piglets (#4 and #6), and these were considered WT. Seven piglets had indel mutations (+ 1, + 3, or − 10 bp) with mutation frequencies of 11.7–89.2%, and these were considered heterogeneous mutants, including mosaic and monoallelic mutations. Among piglets carrying mutations, only one piglet (#2) had three sequence types, including the WT sequence, in the targeted region of *MSTN*, whereas the other mutant piglets had only one kind of edited sequence and WT sequences. No piglets carrying homozygous mutations were identified. Additionally, we have performed Sanger sequencing analysis using samples from major organs in stillborn and crushed pigs (#1, #2, and #9) and muscle tissues in the other pigs carrying mutation (#3, #5, #7, and #8) (Fig. 5, Supplementary Fig. S2). Although the mutation frequencies were variable depending on the organs, the results obtained in muscle tissue samples were consistent with those of deep sequencing analysis of the ear biopsies. The distributions of fiber types in skeletal muscle tissues of one WT (#4) and two piglets carrying heterogeneous mutation with WT (#3 and #5) were investigated (Fig. 6A). Based on immunofluorescence staining of skeletal muscles, we found that the proportion of slow-type myofibers was significantly lower (*p* < 0.05) in *MSTN*-edited piglets than in WT piglets (Fig. 6B). Furthermore, MSTN was quantified in the muscle tissue from WT (#4) and three piglets carrying heterogeneous mutation with WT (#5, #7 and #8) by enzyme-linked immunosorbent assay (ELISA), confirming that it was downregulated in *MSTN*-mutant pigs depending on the mutation rate (Fig. 6C).

Finally, we investigated off-target cleavage in three candidate sites (Supplementary Fig. S3) in WT (control) and delivered piglets by deep sequencing analysis (Table 1). These candidate regions were amplified and indexed using DNA extracted from ear biopsy samples, and NGS analysis was performed in the same manner as for the on-target analysis. DNA extracted from pig samples obtained from the slaughterhouse, where we collected the ovaries for generating gene edited embryos, was used as a control for off-target analysis. The frequencies of unmodified sequences (97.85–98.99%) at the candidate sites in delivered piglets were similar to those in the control pigs (97.85–98.84%), indicating there were no off-target events in gene-edited offspring.

**Table 1:** Frequencies of unmodified sequences at candidate off-target sites analyzed by deep sequencing^a^.

Piglet	MSTN_OT1 (%)	MSTN_OT2 (%)	MSTN_OT3 (%)
Control^b^	22,986/23,492 (97.85)	27,329/27,649 (98.84)	22,216/22,512 (98.69)
#1	17,469/17,785 (98.22)	18,918/19,165 (98.71)	13,355/13,495 (98.96)
#2	20,386/20,833 (97.85)	18,798/19,049 (98.68)	16,906/17,143 (98.62)
#3	20,346/20,739 (98.11)	19,799/20,038 (98.81)	18,951/19,193 (98.74)
#4	22,710/23,187 (97.94)	18,717/18,928 (98.89)	19,321/19,592 (98.62)
#5	19,655/20,030 (98.13)	21,168/21,429 (98.78)	19,918/20,152 (98.84)
#6	17,406/17,702 (98.33)	21,302/21,529 (98.95)	12,042/12,167 (98.97)
#7	17,653/17,978 (98.19)	20,003/20,250 (98.78)	20,341/20,550 (98.98)
#8	19,605/20,006 (98.0)	21,832/22,117 (98.71)	11,236/11,351 (98.99)
#9	24,726/25,196 (98.13)	25,132/25,430 (98.83)	19,907/20,157 (98.76)

^a^The frequency was defined as the ratio of the number of unmodified reads to the total number of aligned reads.

^b^A DNA sample derived from a wild-type pig, not subjected to any gene editing technique, was used as the control.

## Discussion

We generated *MSTN* gene-edited pigs using a novel lipofection-mediated gene-editing strategy during embryogenesis. First, we determined the concentration of lipofection reagent for the efficient delivery of RNPs into embryos. Although the concentration of jetCRISPR did not significantly affect the number of embryos that developed into blastocysts, biallelic mutations were only found in blastocysts derived from embryos treated with 2 µL of jetCRISPR. The lipid transfection reagent surrounds the Cas9 protein and negatively charged gRNA to allow passage into the positively charged cell membrane. During the transfection process, (+/−) charge ratios, resulting from the additional DNA, affect liposome size and lipofection efficiency^30^. Optimization of the transfection reagent-to-RNP ratio is required for efficient transfection and is considered the first step towards the improvement of lipofection-mediated gene targeting efficiency in porcine zygotes and embryos. We also established the versatility of the method by evaluating the gene-editing efficiency for various target genes. The low editing efficiency of the gRNA targeting *KDR* can be improved by the optimization of the gRNA sequence, as demonstrated in our previous study targeting other genes using electroporation^11,31^. Lipofection-mediated gene editing during embryogenesis can be used at various target sites.

To further analyze the mutation efficiency in resulting piglets, we transferred blastocysts treated with RNP transfection reagent into the uterus of a synchronized recipient gilt, and nine piglets were delivered. Deep sequencing revealed that 77% of the piglets (7/9) carried insertions or deletions (indels) in *MSTN*. Immunofluorescence staining of skeletal muscles indicated that the proportion of slow-type myofibers was lower in *MSTN*-edited piglets. *MSTN* regulates the fiber‐type distribution in two ways: (1) by decreasing the proliferation or differentiation of primary fetal myoblasts, leading to a reduction in the number of slow fibers, or (2) by increasing the proliferation or differentiation of secondary fetal myoblasts, resulting in an increase in the number of fast fibers^32^. *MSTN* biallelic mutant pigs show a higher proportion of fast-type myofibers and a relatively low proportion of slow-type myofibers^11^. Furthermore, *MSTN* heterozygous mutant pigs have low *MSTN* mRNA levels and a high proportion of fast-type fibers^33,34^. Furthermore, we quantified MSTN protein concentration in the longissimus dorsi muscle and demonstrated the down regulation of MSTN expression in *MSTN*-mutant pigs. Our finding corroborates that of a previous study wherein the inactivation of MSTN downregulated MSTN expression^35^. Our immunohistological analysis and quantification of MSTN protein concentration in the sleketal muscle indicated that lipofection-mediated gene editing results in the successful downregulation of *MSTN* function.

We also demonstrated that lipofection-mediated RNP introduction can induce mutations without detectable off-target events. Limiting the dose or exposure time of the active gene-editing complex to the target genome is an effective approach to minimize the frequency of off-target cleavage^36^. The RNP-based transfection method has a limited time window for functional on-target gene editing due to the early peak of Cas9 levels after a few hours post-transfection and the more rapid decrease compared with that for Cas9 expression plasmids or *Cas9* mRNA^16^. We demonstrated that the lipofection-based technique as a new introduction method of RNPs into embryos enables efficient one-step gene editing without off-target events in porcine zygote/embryos, similar to somatic cells.

We determined the concentration of lipofection reagent with respect to the gene-editing efficiency and biallelic cleavage; however, the efficiency of lipofection-mediated gene editing was still insufficient compared with that of microinjection- and electroporation-meditated gene-editing^11,26–29^ (Supplementary Fig. S1). None of the delivered piglets carried a biallelic mutation in the *MSTN* gene. Some gene-edited piglets carried more than two different alleles (#2) or extreme deviations in allele frequencies (#1, #8). The genotyping analysis of major organs and muscle tissues also detected multiple alleles, suggesting that the resulting piglets contained mosaic mutants that could be caused by several factors in gene editing for IVF-derived embryos, such as the delayed or persistent activity of RNPs^37^. The onset of the S-phase, the phase of the cell cycle in which DNA is replicated, in the male pronucleus of porcine zygotes is 9–12 h following intracytoplasmic sperm injection^38^. Other studies using monkeys have suggested that mono-allelic mutants can also be associated with the persistent expression and activity of Cas9 in zygotes after the one-cell stage, as it can cause DNA cleavage at later stages of embryonic development^39,40^. In this study, RNP was introduced at 29 h from the start of insemination, and at this point, some of the embryos have already reached the genome replication phase and entered the first cell division, explaining the mosaicism. In contrast, we demonstrated that the lipofection-mediated introduction of RNP during IVF is insufficient for practical gene editing^17^. In mice, major zygotic genome activation with an open chromatin state occurs at the 2-cell stage^41,42^. On the other hand, major genome activation has been detected at the 4-cell stage in porcine embryos^43,44^. The open chromatin state may improve the accessibility of the CRISPR system to the target site, and the duration of the target residence of CRISPR/Cas9 is correlated with cleavage activity^45^. Under the present conditions, lipofection treatment after the start of genome replication is essential for efficient gene editing; a substantial limitation of our lipofection-mediated gene editing system is high mosaicism. To achieve highly efficient gene editing without mosaicism, the timing of lipofection-mediated introduction of the CRISPR/Cas9 system should be further optimized.

Another limitation of lipofection-mediated gene editing is the necessity of ZP removal. Removal of ZP did not affect embryonic development, however aggregation of ZP-free embryos is another factor that caused genetic mosaicism in this study. We used a group culture system of ZP-free embryos after lipofection, which could cause aggregation of two or more embryos and result in chimeric blastocysts/piglets. Deep sequencing analysis detected these chimeric piglets as genetic mosaics, which also decreased the biallelic mutation rates. Furthermore, aggregation of ZP-free embryos suggests the possibility of generating male–female chimeras. In a previous study where Day 6 or Day 7 inner cell mass (ICM) cells were injected into Day 6 blastocysts, it was demonstrated that the resulting chimera pig had a male phenotypic sex, and the chimeric pigs were all fertile^46^. In this study, all of resulting pigs were male. These results suggest the possibility of aggregated male–female chimeras. Male–female chimerism may also affect fertility and this is a serious limitation of our group culture system. However, this study aimed to evaluate whether lipofection-mediated gene editing is able to generate gene-edited live pigs. Therefore, male–female chimerism was not analyzed as this would be outside the scope of the study. Thus, an individual culture system of lipofection-treated ZP-free embryos with highly efficient embryonic development should be optimized for generating biallelic mutants without chimerism.

Furthermore, we did not evaluate the effects of the Cas9 concentration on lipofection-mediated gene editing. We have previously demonstrated that the elevation of the Cas9 concentration improves the rate of blastocysts carrying biallelic mutations and the gene-editing efficiency in the resulting blastocysts with the CRISPR/Cas9 system introduced by electroporation^28,47^. Increasing the RNP concentration during lipofection-mediated gene editing has the potential to improve the gene-editing efficiency. Recent studies have suggested highly efficient Cas9 variants, such as Cas9-HF1^48^, evo-Cas9^49^, eSpCas9^50^, and Hypa-Cas9^51^. The use of these variants may also improve the gene-editing efficiency.

In conclusion, we successfully established a novel method for generating genetically modified pigs via transfection of the CRISPR/Cas9 system into porcine embryos using RNP transfection reagents. Lipofection-mediated gene editing in embryos is a feasible system for use with ZP-free oocytes/embryos; however, the method is in the initial stage of development and numerous issues remain to be resolved; these include insufficient gene-editing efficiency of treated embryos and resulting pigs, and the necessity for repeated trials including embryo transfer to evaluate the effects of lipofection treatment on pregnancy.

## Methods

### Animals

Animal husbandry and procedures of anesthesia/euthanasia were performed as described previously^27^. One sexually mature Landrace gilt was obtained from the Tokushima Prefectural Livestock Research Institute (Tokushima, Japan), housed in a temperature-controlled room (25 ± 3 °C) under a 12-h light/12-h dark cycle with free access to water, and provided with commercial feed (JA Nishinihon Kumiai Shiryou, Hyogo, Japan). The health condition of each pig was observed daily by the animal husbandry staff under the supervision of an attending veterinarian. To minimize animal suffering, all surgical procedures were performed under anesthesia by intramuscular injection of 10 mg/kg ketamine (Ketalar, ketamine hydrochloride, Daiichi Sankyo Pharmaceutical, Tokyo, Japan) and continuous inhalation of 2–3% isoflurane (Mylan, Osaka, Japan) in the operating room. Euthanasia was performed by intravenous injection of a potassium chloride solution (3 mmol/kg) under deep anesthesia by isoflurane according to the American Veterinary Medical Association Guidelines for the Euthanasia of Animals.

### Design of gRNA sequence

Alt-R CRISPR crRNAs and tracrRNA system purchased from IDT was used as gRNA. The gRNAs were designed using the CRISPR direct web tool (https://crispr.dbcls.jp/)^52^. To minimize off-target effects, the 14 nucleotides at the 3′ end of the designed gRNAs only matched the target regions of each genes and had no other sequence matches in the pig genome, as determined using the COSMID web tool (https://crispr.bme.gatech.edu/)^53^.

### Oocyte collection, in vitro maturation, and fertilization

Oocyte collection, IVM, and IVF were performed as described previously^54^. Briefly, pig ovaries were obtained from prepubertal crossed gilts (Landrace × Large White × Duroc breeds) at a local slaughterhouse. Cumulus-oocyte complexes (COCs) were collected from ovaries and cultured in maturation medium at 39 °C in a humidified incubator containing 5% CO_2_. The matured oocytes were subjected to IVF. frozen-thawed ejaculated spermatozoa were transferred into 5 mL of fertilization medium (PFM; Research Institute for the Functional Peptides Co.) and washed by centrifugation at 500×*g* for 5 min. The pelleted spermatozoa were resuspended in fertilization medium and adjusted to a density of 1 × 10^6^ cells/mL. Approximately 50 oocytes were transferred to 500 µL of sperm-containing fertilization medium, covered with mineral oil in 4-well dishes, and co-incubated for 5 h at 39 °C in a humidified incubator containing 5% CO_2,_ 5% O_2_, and 90% N_2_. After co-incubation, the attached spermatozoa were gently removed from the oocytes by mechanical pipetting. The putative zygotes were transferred to PZM-5 and cultured for 24 h until RNP transfection.

### ZP removal and RNP transfection

Embryos at 1- to 8-cell stages collected at 29 h from the start of IVF were exposed to 0.5% (w/v) actinase-E in Dulbecco’s phosphate-buffered saline (Nissui Pharmaceutical, Tokyo, Japan) for 20–30 s, transferred to PZM-5 without actinase-E, and freed completely from their ZP by gentle pipetting. The ZP-free embryos were incubated at 39 °C in a humidified incubator containing 5% CO_2_, 5% O_2_, and 90% N_2_ for 1 h before the reagent-mediated introduction of the CRISPR/Cas9 system using jetCRISPR.

RNP transfection solution was prepared by adding 0.5, 1, or 2 μL of jetCRISPR to the nucleic acid-free duplex buffer (IDT) containing RNP complex prepared by mixing gRNA (Supplementary Table S1) and Cas9 protein at a final concentration of 167 ng/μL and 500 ng/μL, respectively, to make a final volume of 30 μL. After 15 min of incubation at 25 °C, the RNP transfection solution was added to 470 μL of PZM-5 containing ZP-free embryos and then co-incubated for 5 h in a humidified incubator containing 5% CO_2_, 5% O_2_, and 90% N_2_. After 5 h of incubation, ZP-free embryos were washed and cultured in PZM-5 for 2 days. Subsequently, the embryos were cultured in porcine blastocyst medium (PBM; Research Institute for the Functional Peptides Co.) for 4 days to evaluate their ability to develop to the blastocyst stage and the genotype of resulting blastocysts. As a control for the analysis of embryonic development, some ZP-free embryos without RNP transfection were cultured in the same manner. ZP-free embryos were cultured together, because single culture of ZP-free embryos showed decreased blastocyst formation rates in our culture conditions.

### Electroporation

Electroporation was performed as described previously^11^. Briefly, an electrode (LF501PT1-20; BEX, Tokyo, Japan) was connected to a CUY21EDIT II electroporator (BEX) and was set under a stereoscopic microscope. The inseminated ZP-intact 50 zygotes collected at 10 h from the start of IVF were washed with Opti-MEM I solution (Gibco/Invitrogen, Carlsbad, CA, USA) and were placed in a line in the electrode gap in a chamber slide filled with 10 μL of Nuclease-Free Duplex Buffer (IDT) containing 100 ng/μL gRNA targeting *KDR* and 100 ng/μL Cas9 protein (Takara Bio). After electroporation (five 1-ms square pulses at 25 V), the zygotes were washed with PZM-5 and were cultured until embryo transfer (for 12 h) or for 3 days. The embryos that were cultured for 3 days were subsequently incubated in PBM for 4 days, and resulting blastocysts were used for genotyping analysis. Zygotes and embryos were incubated at 39 °C in a humidified incubator containing 5% CO_2_, 5% O_2_, and 90% N_2_.

### Analysis of the targeted gene in embryos

Analysis of the targeted gene in embryos was performed as described previously^27^. Genomic DNA was isolated from blastocysts by boiling in a 50 mM NaOH solution. After neutralization, the DNA samples were subjected to polymerase chain reaction (PCR) using KOD One PCR Master Mix (Toyobo, Osaka, Japan) according to the manufacturer’s instructions using specific primers (Supplementary Table S1). The PCR products were extracted by agarose gel electrophoresis using a Fast Gene Gel/PCR Extraction Kit (Nippon Genetics, Tokyo, Japan). The PCR products were directly sequenced by Sanger sequencing using the BigDye Terminator Cycle Sequencing Kit (version 3.1; Thermo Fisher Scientific K.K., Tokyo, Japan) and an ABI 3500 genetic analyzer (Applied Biosystems, Foster City, CA, USA). The TIDE bioinformatics package was used to determine the genotype of each blastocyst^25^. Genotypes of blastocysts were classified as homozygous editing (including only single types of editing), heterozygous editing without WT (including multiple types of editing but carrying no WT sequences), heterogeneous editing with WT (including mosaic or heterozygous mutation carrying more than one type of mutation and the WT sequence, and monoallelic mutation), or WT (carrying only the WT sequence). The editing rate was defined as the ratio of the number of gene-edited blastocysts to the total number of sequenced blastocysts. Editing efficiency was defined as the proportion of indel mutation events in mutant blastocysts.

### Embryo transfer

Recipient gilt, after synchronization of estrous cycles, was prepared for embryo transfer as described previously^55^. In brief, 0.2 mg of cloprostenol (Planate; MSD Animal Health, Tokyo, Japan) was administered by intramuscular injection to pregnant gilt 4–7 weeks after mating. Subsequently, a second intramuscular injection of 0.2 mg of cloprostenol and 1000 IU of eCG (PMSG, ZENOAQ, Fukushima, Japan) was administered to the gilt 24 h after the first injection of cloprostenol. At 72 h after the intramuscular injection of eCG, 1500 IU of hCG (Gestron 1500, Kyoritsu Seiyaku, Tokyo, Japan) was administered to the gilt. Approximately 125 h after the hCG intramuscular injection, early blastocysts derived from embryos treated with the RNP transfection reagent were transferred into the uterus of a recipient gilt under anesthesia.

### Mutation analysis in piglets by deep sequencing and Sanger sequencing

Genomic DNA was isolated from ear biopsies by boiling in a 50 mM NaOH solution. After neutralization, the genomic regions flanking the gRNA target sequences were amplified by two-step PCR using specific primers and the index PCR primers following the manufacturer’s instructions (Illumina, Hayward, CA, USA) (Supplementary Table S2). After gel purification, the amplicons were subjected to MiSeq sequencing using the MiSeq Reagent Kit v. 2 (250 cycles) (Illumina, San Diego, CA, USA). CRISPResso2^56^ was used for data analysis. The genotypes of piglets were classified according to the definition of genotypes in embryos described above.

Genomic DNA was isolated from ear, muscle, lung, heart, liver, and kidney by boiling in 50 mM NaOH. After neutralization, the DNA samples were subjected to PCR using specific primers targeting *MSTN* (Supplementary Table S1). The PCR products were extracted by agarose gel electrophoresis and subjected to Sanger sequencing as described above.

### Off-target effects determined by deep sequencing

An off-target analysis was performed as described previously^11^. The COSMID webtool was used to predict off-target candidates^53^. The genomic regions flanking potential off-target sites were amplified by two-step PCR using specific primers (Supplementary Table S3) and analyzed by a MiSeq sequencing analysis, as described above. Indels or substitutions were measured within a 5-bp window around the predicted Cas9 cleavage site in each off-target site. A small number of amplicons carrying different sequences that were also detected in the WT sample were considered as sequencing errors.

### Immunofluorescence staining

Longissimus dorsi muscle biopsy samples obtained from the 40-day-old piglets were fixed in a 4% paraformaldehyde neutral-buffered solution (Wako, Osaka, Japan) and manually embedded in paraffin. To analyze the distribution of skeletal muscle fiber types, paraffin-embedded sections were deparaffinized and antigen retrieval was performed by autoclaving the slides in citrate buffer (pH 6.0) for 15 min. Slow and fast myofibers were detected using mouse anti-slow skeletal muscle myosin (ab11083, 1/500; Abcam, Cambridge, UK) and rabbit anti-fast skeletal muscle myosin (ab91506, 1/500; Abcam), respectively. The sections were subsequently incubated for 2 h at 25 °C with Alexa Fluor 594 goat anti-mouse IgG (ab150116, 1/500; Abcam) and Alexa Fluor 488 goat anti-rabbit IgG (ab150077, 1/500; Abcam). After staining, seven images were obtained per sample using a BZ-X710 microscope (KEYENCE, Osaka, Japan), and the slow and fast muscle fiber areas were calculated using BZ-X Analyzer (KEYENCE). The percentage of slow myofibers was defined as the percentage of slow myofibers to the sum of the slow and fast myofiber areas.

### Quantifying muscle MSTN protein concentration

Longissimus dorsi muscle biopsy samples were obtained from 6-month-old pigs under anesthesia. MSTN protein concentration was determined using an ELISA kit (R&D Systems, Minneapolis, MN, USA). Total protein extraction from muscle samples and the quantification of MSTN protein concentration were performed according to the manufacturer's instructions. The concentration of all protein extracts were quantified using the BCA protein assay kit (Takara Bio). The samples were diluted to 1.0 mg mL^−1^ concentration before starting the assay.

### Statistical analyses

Data for blastocyst formation and mutation efficiencies were evaluated using analysis of variance (ANOVA) followed by protected Fisher’s least significant difference tests using StatView (Abacus Concepts, Berkeley, CA, USA). All percentage data were subjected to arcsine transformation before ANOVA. The percentage of mutated blastocysts was analyzed using chi-squared tests with Yates’ correction. Differences with a *p* value of ≤ 0.05 were considered statistically significant.

### Study approval

The animal experiments were approved by the Institutional Animal Care and Use Committee of Tokushima University (approval number: T2019-11). All animal care and experimental procedures were performed in accordance with the Guidelines for Animal Experiments of Tokushima University and in compliance with the ARRIVE guidelines.

## Supplementary Information

Supplementary Information.

## Data Availability

The data presented in this study are available on request from the corresponding author. The data are not publicly available to preserve privacy of the data.
