# Supplementary material for: Generation of mutant pigs by lipofection-mediated genome editing in embryos
Source: Sci Rep. 2021 Dec 13;11:23806. doi: 10.1038/s41598-021-03325-5 (PMC8668999; doi:10.1038/s41598-021-03325-5)
Supplement: Supplementary file 1 — Supplementary Information. [file 41598_2021_3325_MOESM1_ESM.docx]

**Supplementary information**

**Generation of mutant pigs by lipofection-mediated genome editing in embryos**

Maki Hirata^1,2^, Manita Wittayarat^3^, Zhao Namula^1,4^, Quynh Anh Le^1^, Qingyi Lin^1^, Koki Takebayashi^1^, Chommanart Thongkittidilok^1^, Taro Mito^1,2^, Sayuri Tomonari^1^, Fuminori Tanihara^1^* and Takeshige Otoi^1,2^

^1^Faculty of Bioscience and Bioindustry, Tokushima University, Tokushima, Japan

^2^Bio-Innovation Research Center, Tokushima University, Tokushima, Japan

^3^Faculty of Veterinary Science, Prince of Songkla University, Songkhla, Thailand

^4^College of Coastal Agricultural Sciences, Guangdong Ocean University, Guangdong, China

*Correspondence: f_tanihara@jichi.ac.jp; Tel.: +81-285-58-7490 (Current address: Center for Development of Advanced Medical Technology, Jichi Medical University, Tochigi, Japan)

**
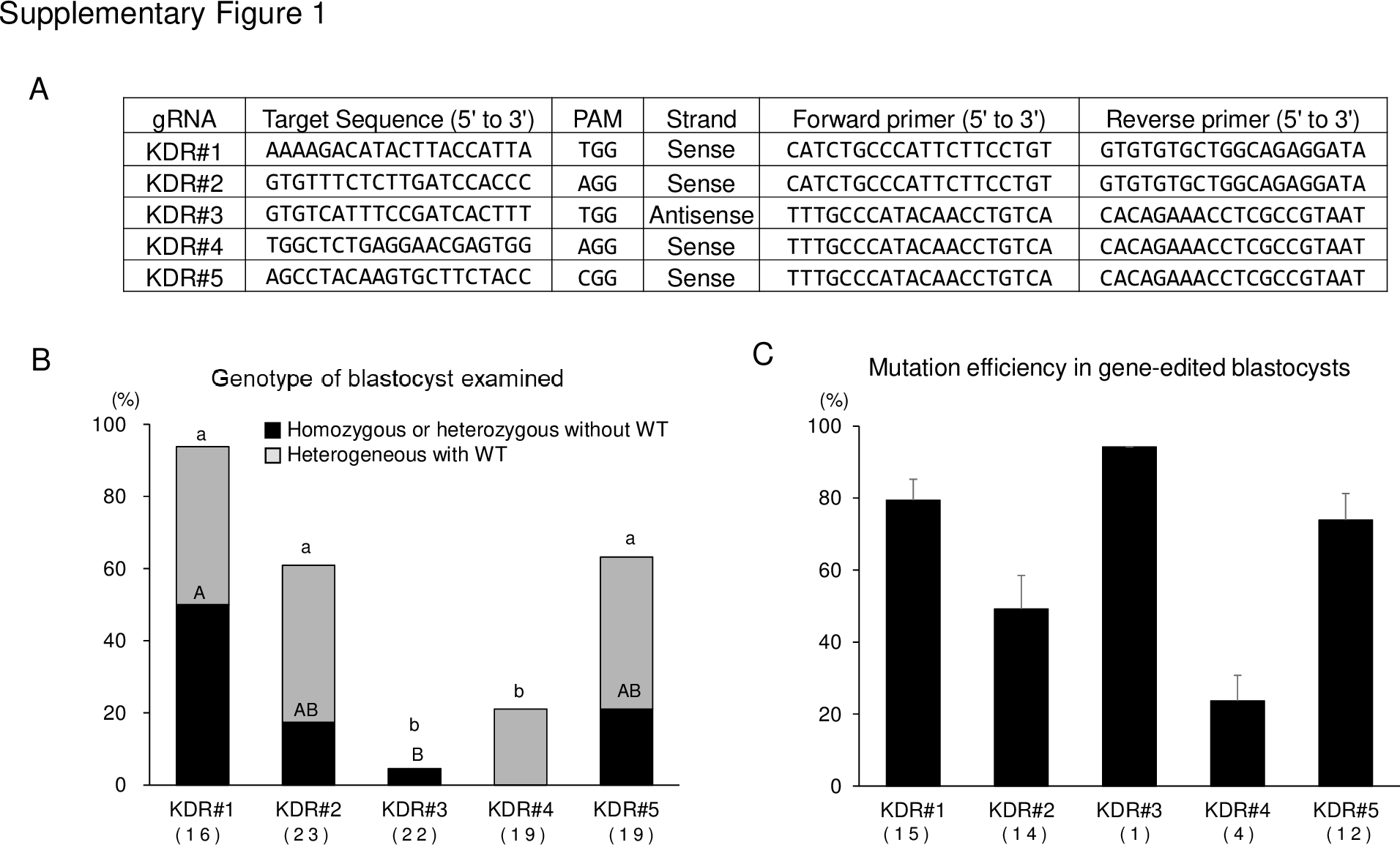
**

**Supplementary Figure S1. Confirmation of electroporation-mediated gene editing targeting *KDR*.** Five gRNAs were designed and gRNA/Cas9 complex was introduced into *in-vitro* fertilized zygotes using electroporation. (A) gRNA sequences targeting *KDR* and the genomic structure of the *KDR* locus. (B) Frequency of gene editing in the target regions of blastocysts derived from the embryos introduced Cas9 protein and gRNA by electroporation. Gene editing of blastocysts was determined by Sanger sequencing and TIDE. The percentage of genetically edited blastocysts was defined as the ratio of the number of gene-edited blastocysts to the total number of blastocysts examined. (C) Mutation efficiency in gene-edited blastocysts. Editing efficiency was defined as the proportion of indel mutation events in blastocysts carrying mutations. Homozygous or heterozygous without WT: blastocysts carrying single types of editing or multiple types of editing but no WT sequences; Heterogeneous with WT: blastocysts carrying mosaic mutation or heterozygous mutation carrying more than one type of mutation and the WT sequence, and monoallelic mutation. Each bar represents the mean ± SEM. Four replicate trials were performed and the numbers in parentheses indicate the total number of blastocysts examined (B and C). Percentages of blastocysts carrying mutations in target genes were analyzed using chi-squared tests (B). ^a–b, A-B^Values with different superscripts differ significantly (*p* < 0.05) and labels containing the same letter mean no significant difference.

**
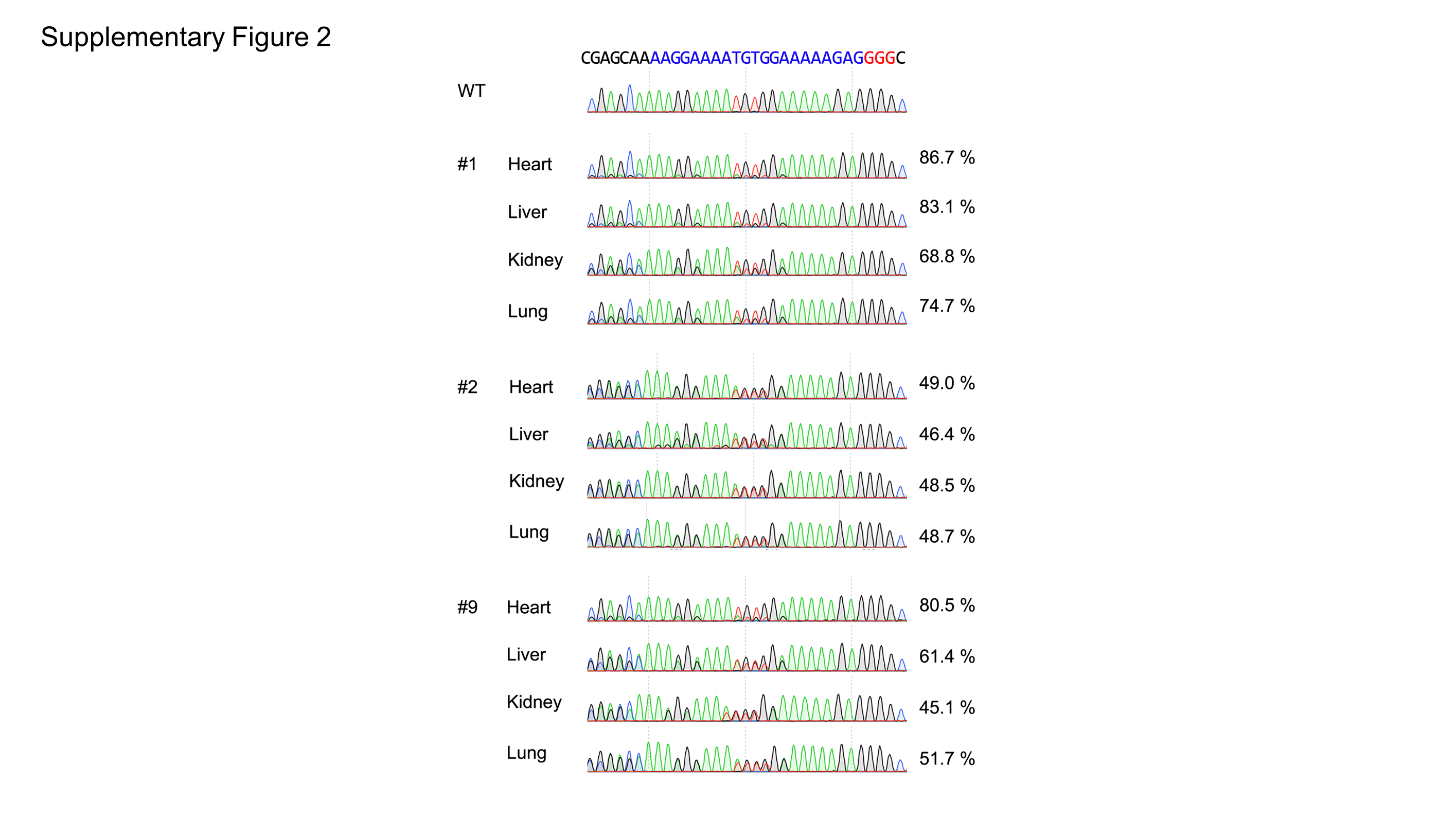
**

**Supplementary Figure S2. Sanger sequencing analysis of major organs derived from MSTN mutant pigs.** Tissue samples from major organs were collected from stillborn and crushed pigs. Total efficiency is defined as the frequency of indel mutations decomposed from Sanger sequence data by TIDE analysis.


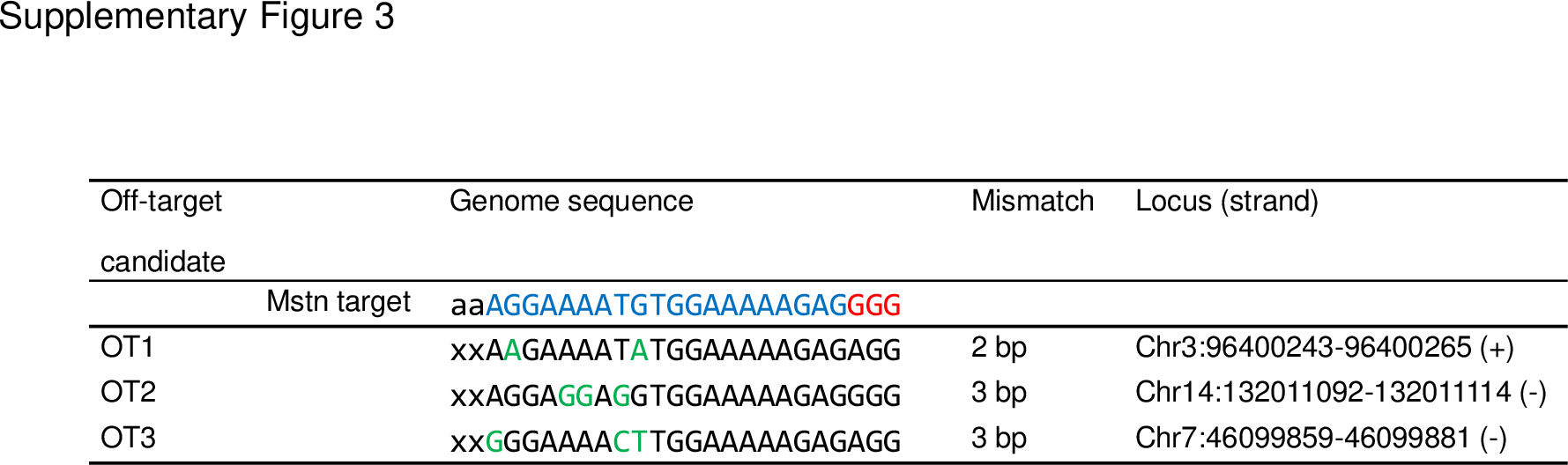


**Supplementary Figure S3. Genome sequences and positions of possible off-target sites.** Nucleotides in blue and red represent the target sequences and the PAM sequences of *MSTN* gRNA, respectively. Nucleotides in green represent mismatches with the gRNA sequence.

**Supplementary Table S1. gRNA and primer sequences targeting five genes.**

| Target gene | Target sequence (5' to 3') | PAM | Strand | Forward primer (5' to 3') | Reverse primer (5' to 3') |
| --- | --- | --- | --- | --- | --- |
| *MSTN* | AGGAAAATGTGGAAAAAGAG | GGG | Sense | ATGCAAAAACTGCAAATCTATG | TGTAGGCATGGTAATGATCG |
| *B4GALNT2* | GTCTCCTCAGGTTCACTGCG | GGG | Antisense | TAGGGGGAAAAACACACTGG | CACCCTCGGGAATGAGTAGA |
| *KDR* | AAAAGACATACTTACCATTA | TGG | Sense | CATCTGCCCATTCTTCCTGT | GTGTGTGCTGGCAGAGGATA |
| *PDX* | TGGCGAGGAGCAGTACTACG | CGG | Sense | ATAGAAGTCCAAATATTTTCCCCGC | ACCTCGTACGGGGAGATGTC |
| *CMAH* | GAAGCTGCCAATCTCAAGGA | AGG | Sense | GCTGTCAATGCTCAGGGATT | TGCCAAACCTAATTGGGAGA |
| *GGTA1* | AGACGCTATAGGCAACGAAA | AGG | Sense | AAAAGGGGAGCACTGAACCT | CCTGTCGGGAATGTTCTCAT |

**Supplementary Table S2. Oligonucleotide sequences for the validation of introduced mutations in piglets by deep sequencing**

| Primer | | Common sequence | |  | Specific sequence |
| --- | --- | --- | --- | --- | --- |
| Forward | | ACACTCTTTCCCTACACGACGCTCTTCCGATCT | |  | TTGGCGTTACTCAAAAGCAA |
| Reverse | | GTGACTGGAGTTCAGACGTGTGCTCTTCCGATCT | |  | CTGTCATCTCTCTGGACATCG |
|  | |  |  |  |  |

**Supplementary Table S3. Oligonucleotide sequences used for an off-target analysis by deep sequencing**

| Primer |  | Off-target candidate | Common sequence |  | Specific sequence |
| --- | --- | --- | --- | --- | --- |
| Forward |  | OT1 | ACACTCTTTCCCTACACGACGCTCTTCCGATCT |  | GCTCCATTAGACCCCTAGCC |
|  |  | OT2 |  |  | GCGTGGACAGTTCTTCTGGT |
|  |  | OT3 |  |  | TCCTGAGTGTCCTCTTTGCTT |
| Reverse |  | OT1 | GTGACTGGAGTTCAGACGTGTGCTCTTCCGATCT |  | AGAGGCAAATCATCCACAGC |
|  |  | OT2 |  |  | ACATGAGCTGGGTCATGAAT |
|  |  | OT3 |  |  | GAAATTTGGTTGAGGCCAGA |
